# Synergistic Combination of Antimicrobial Peptides and Cationic Polyitaconates in Multifunctional PLA Fibers

**DOI:** 10.1021/acsabm.3c00576

**Published:** 2023-10-20

**Authors:** Alberto Chiloeches, Jakub Zágora, Daniela Plachá, Marcelo D. T. Torres, Cesar de la Fuente-Nunez, Fátima López-Fabal, Yolanda Gil-Romero, Raquel Fernández-García, Marta Fernández-García, Coro Echeverría, Alexandra Muñoz-Bonilla

**Affiliations:** † Instituto de Ciencia y Tecnología de Polímeros (ICTP-CSIC), C/Juan de la Cierva 3, Madrid 28006, Spain; ‡ Universidad Nacional de Educación a Distancia (UNED), C/Bravo Murillo 38, Madrid 28015, Spain; § Nanotechnology Centre, CEET, VSB—Technical University of Ostrava, 17. Listopadu 2172/15, Ostrava-Poruba 708 00, Czech Republic; ∥ Machine Biology Group, Departments of Psychiatry and Microbiology, Institute for Biomedical Informatics, Institute for Translational Medicine and Therapeutics, Perelman School of Medicine, University of Pennsylvania, Philadelphia, Pennsylvania 19104, United States; ⊥ Departments of Bioengineering and Chemical and Biomolecular Engineering, School of Engineering and Applied Science, University of Pennsylvania, Philadelphia, Pennsylvania 19104, United States; # Penn Institute for Computational Science, University of Pennsylvania, Philadelphia, Pennsylvania 19104, United States; ¶ Hospital Universitario de Móstoles C/Dr. Luis Montes, s/n, Móstoles 28935, Madrid, Spain; ∇ Facultad de Ciencias Experimentales, Universidad Francisco de Vitoria, Carretera Pozuelo a Majadahonda, Km 1.800, Madrid 28223, Spain

**Keywords:** cationic polymers, antimicrobial peptide, fibers, supercritical impregnation, antimicrobial

## Abstract

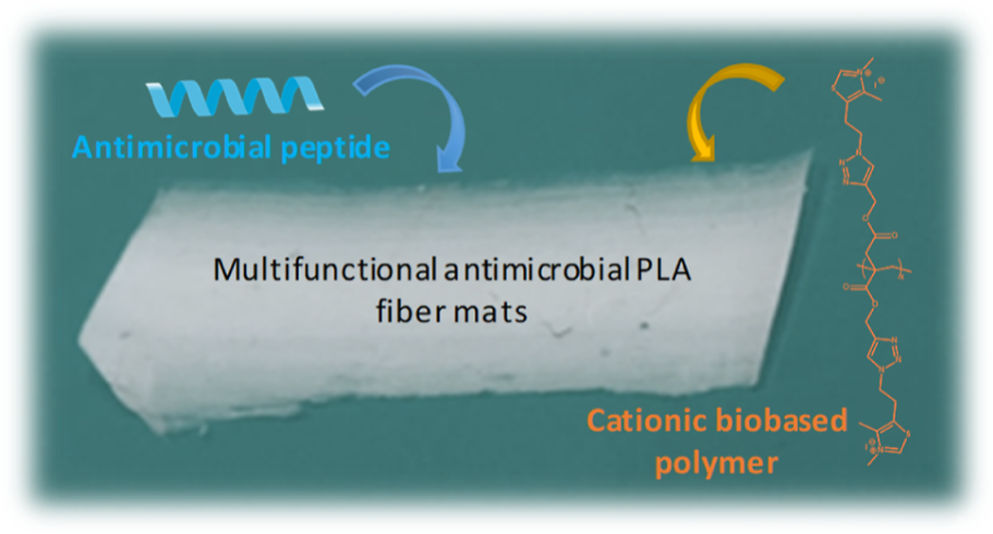

Combining different antimicrobial agents has emerged as a promising strategy to enhance efficacy and address resistance evolution. In this study, we investigated the synergistic antimicrobial effect of a cationic biobased polymer and the antimicrobial peptide (AMP) temporin L, with the goal of developing multifunctional electrospun fibers for potential biomedical applications, particularly in wound dressing. A clickable polymer with pendent alkyne groups was synthesized by using a biobased itaconic acid building block. Subsequently, the polymer was functionalized through click chemistry with thiazolium groups derived from vitamin B1 (PTTIQ), as well as a combination of thiazolium and AMP temporin L, resulting in a conjugate polymer–peptide (PTTIQ-AMP). The individual and combined effects of the cationic PTTIQ, Temporin L, and PTTIQ-AMP were evaluated against Gram-positive and Gram-negative bacteria as well as Candida species. The results demonstrated that most combinations exhibited an indifferent effect, whereas the covalently conjugated PTTIQ-AMP displayed an antagonistic effect, potentially attributed to the aggregation process. Both antimicrobial compounds, PTTIQ and temporin L, were incorporated into poly(lactic acid) electrospun fibers using the supercritical solvent impregnation method. This approach yielded fibers with improved antibacterial performance, as a result of the potent activity exerted by the AMP and the nonleaching nature of the cationic polymer, thereby enhancing long-term effectiveness.

## 1 Introduction

The utilization of antimicrobial and biodegradable compounds derived from renewable resources has gained significant importance in the medical field. Research and development efforts are increasingly focused on materials obtained from sustainable sources. Incorporating antimicrobial biobased polymers into biomaterials offers a promising approach to reduce microbial contamination and decrease reliance on antibiotics while maintaining biodegradability and sustainability. This is particularly crucial for single-use items like face masks and wound dressings.^1^ Electrospinning has emerged as a well-established and versatile technology for producing submicron-sized fibers, which provides numerous advantages such as a high surface-to-volume ratio. These electrospun fiber mats serve as physical barriers, preventing microbial penetration while facilitating oxygen and gas transfer, especially important for wound dressing applications.^2^ Moreover, the high surface area of these fiber mats enables the incorporation of bioactive molecules, including antimicrobial compounds, thereby reducing the risk of infections.

In various biomedical applications, including wound dressings, biobased and biodegradable polymers with antimicrobial properties have been successfully incorporated into electrospun fibers.^3−7^ Generally, these antimicrobial polymers possess cationic characteristics and exert their antimicrobial activity through contact interactions rather than release-based strategies. This mechanism involves electrostatic interactions with microbial membranes, leading to physical damage to the bacterial wall. Consequently, this approach minimizes the likelihood of the development of bacterial resistance.

Antimicrobial peptides (AMPs) have emerged as a highly promising class of antibacterial agents due to their low propensity for inducing resistance. These peptides exhibit efficacy against a broad spectrum of microorganisms, including both planktonic bacteria and biofilms.^8−11^ AMPs achieve their antimicrobial activity by disrupting the cell membranes of bacteria, a mechanism facilitated by their unique physicochemical properties and amphipathic characteristics. These attributes make AMPs ideal candidates for incorporation into materials designed for various biomedical applications,^12,13^ having been incorporated in fibrous scaffolds to fabricate antimicrobial wound dressings.^14−16^ An example of an AMP family is the temporins, which are derived from the skin of the frog *Rana temporia* and have shown potential for topical applications.^17,18^ Temporins are short peptides with a low net positive charge and demonstrate effectiveness against both Gram-positive and Gram-negative bacteria, viruses, and *Candida* spp. Among the approximately 130 isoforms within this family, temporin L stands out as a particularly promising candidate due to its potent antimicrobial activity (ranging from 0.3 to 3.6 μM for different bacterial strains^19^). At physiological pH, the cationic net charge of temporin L, coupled with its amphipathic nature, allows it to adopt an α-helical conformation, facilitating interactions with the phospholipid components of bacterial membranes. Furthermore, synergistic antibacterial effects have been observed when temporin L is combined with temporins A and B.^20^ Notably, investigations combining temporin L with clinically used antibiotics have yielded varied outcomes, ranging from synergy to antagonism.^21^ In this study, we explore the impact of combining AMP temporin L with a cationic polymer on antimicrobial activity.

To investigate the potential synergistic effects of combining antimicrobial compounds, we chose a biobased polyitaconate containing thiazolium groups based on its advantageous properties, including biobased origin, biodegradability, high antimicrobial activity, and low toxicity. Different synthetic approaches were employed to combine this polymer with temporin L. The antimicrobial activity of the compounds was assessed through determination of minimal inhibitory concentration (MIC) values and calculation of the fractional inhibitory concentration (FIC) index. Additionally, to explore the impact of covalently binding the peptide with the polymer, we synthesized a conjugate using click chemistry and examined its antimicrobial properties. Lastly, both antimicrobial compounds were incorporated into electrospun PLA fibers, and their efficacy against bacteria was evaluated.

Antimicrobial agents can be effectively immobilized into the fibers by incorporation into the solution before the generation of the fibers.^15^ However, the processing conditions during electrospinning can significantly impact the stability, activity, and distribution of these compounds within the fibers. The antimicrobials may alter solution properties, such as viscosity or conductivity, which can negatively affect the production of homogeneous fibers. An alternative approach is the direct incorporation of bioactive agents into fibers after the electrospinning process, through either chemical modification reactions or impregnation methods. However, traditional methods often suffer from drawbacks, including the use of toxic organic chemicals, issues of dissolution or compatibility, undesirable reactions and degradation, low loading yields, and heterogeneous incorporation. To address these challenges, supercritical solvent impregnation (SSI) has emerged as a highly advantageous technique for the design of functional materials. This method offers numerous benefits and possibilities while serving as a nontoxic and environmentally friendly alternative, particularly when utilizing carbon dioxide (scCO_2_).^22^ The favorable critical properties of scCO_2_, such as low temperature and critical pressure, along with its nontoxic nature, widespread availability, and ease of removal through decompression, make it an ideal choice for SSI. In this process, the active compound is dissolved in scCO_2_ and brought into contact with the materials to be impregnated. Due to the low viscosity and near-zero surface tension of the supercritical solution, it can easily penetrate the polymer matrix, enabling high impregnation yields. SSI methods have been successfully employed to immobilize various antimicrobial agents, particularly essential oils, into polymeric materials.

In this study, we utilized SSI methods to load the AMP temporin L into electrospun fibers based on poly(lactic acid) (PLA), which also contained a cationic biobased polymer. The objective was to develop multifunctional biobased fibers with antimicrobial activity.

## 2 Experimental Part

### 2.1 Materials

To prepare antimicrobial polyitaconates, we acquired the following chemicals. Itaconic acid (IA, ≥99%), propargyl alcohol (≥99%), hydroquinone (99%), copper chloride (CuCl, ≥99.995%), N,N,N′,N″,N″-pentamethyldiethylenetriamine (PMDETA, 99%), iodomethane (MeI, 99.5%), neutral aluminum oxide, sodium bicarbonate (NaHCO_3_, ≥99.7%), magnesium sulfate anhydrous (MgSO_4_, ≥99.5%), anhydrous tetrahydrofuran (THF, 99.9%), and anhydrous *N*,*N*-dimethylformamide (DMF, 99.8%) were obtained from Merck and used as received. The synthesis of 2-(4-methylthiazol-5-yl)ethanol azide was conducted according to the previously reported method.^23^ 2,2′-Azobis(isobutyronitrile) (AIBN, 98%), used as a radical initiator, was purchased from Acros. Tetrahydrofuran (THF), dimethylformamide (DMF), ethanol (EtOH), hexane, and chloroform (CHCl_3_) were purchased from Scharlau. Ethyl acetate (EtOAc) was procured from Cor Qumica S.L., toluene was purchased from Merck, and sulfuric acid (H_2_SO_4_) was purchased from PanReac. Deuterated solvents for NMR measurements, chloroform (CDCl_3_), and dimethyl sulfoxide (DMSO-*d*_6_), were acquired from Sigma-Aldrich. Cellulose dialysis membranes (CelluSep T1) were purchased from Membrane Filtration Products, Inc., and PLA (6202D) was provided by NatureWorks.

To conduct the antibacterial assays, the following materials were acquired: phosphate buffered saline (PBS) powder, pH 7.4, obtained from Merck and BBL Mueller–Hinton broth purchased from Becton, Dickinson and Company were employed as the bacterial media. 96-well microplates were purchased from BD Biosciences. Columbia agar (5% sheep blood) plates were provided by bioMérieux. Sodium chloride solution (NaCl, bioXtra) was obtained from Merck. *Pseudomonas aeruginosa* (*P. aeruginosa*, ATCC 27853), *Escherichia coli* (*E. coli*, ATCC 25922), *Staphylococcus aureus* (*S. aureus*, ATCC 29213), *Enterococcus faecalis* (*E. faecalis*, ATCC 29212), and *Candida albicans* (*C. albicans*, ATCC 200955) were purchased from Oxoid and used as microbial strains.

### 2.2 Synthesis of Clickable Polymer, P(PrI)

The P(PrI) polymer bearing alkyne groups was synthesized as previously described from biobased itaconic acid.^24^ First, itaconic acid was reacted with propargyl alcohol via a condensation reaction using H_2_SO_4_ as a catalyst under reflux in THF/toluene. After purification, the monomer (PrI) was polymerized by conventional radical polymerization at a total concentration of 2 M in anhydrous DMF, 5 mol % of AIBN initiator, at 70 °C under a nitrogen atmosphere for 24 h. The polymer P(PrI) was purified through precipitation in isopropyl alcohol and subsequently dried under vacuum, resulting in the formation of a white solid (*M*_n_ = 6700 g/mol, *M*_w_/*M*_n_ = 1.6). ^1^H NMR (400 MHz, CDCl_3_, δ, ppm): 4.67 (4H, –CH_2_C=CH), 2.49 (2H, –CH_2_C=C*H*), 1.99–1.00 (8H, CH_2_–CO and –CH_2_–chain).

### 2.3 Synthesis of Thiazolium Derivative, M2Q

2-(4-Methylthiazol-5-yl)ethanol azide (M2) was synthesized from 5-(2-hydroxyethyl)-4-methylthiazole by the formation of the mesylate intermediate followed by reaction with sodium azide.^23^ Subsequently, the obtained azide-functionalized thiazole was subjected to N-alkylation reaction of single methyl iodide, resulting in the formation of the cationic thiazolium derivative (M2Q). Briefly, M2 (2.000 g, 11.8 mol) was dissolved in 25 mL of acetonitrile, and then, a substantial excess of MeI was introduced (110 μL, 17.7 mmol). The reaction mixture was stirred at 90 °C for 24 h. After that, the solvent was partially removed by rotaevaporation, and the thiazolium derivative M2Q was purified by precipitation in ether and dried under vacuum (3.570 g, 97% yield). ^1^H NMR (400 M Hz, DMSO-*d*_6_, δ, ppm): 10.01 (s, 1H, H-thiazole), 4.08 (s, 3H, CH_3_–N+), 3.65 (t, 2H, N_3_–CH_2_–CH_2_–thiazole), 3.18 (t, 2H, N_3_–CH_2_–CH_2_–thiazole), 2.45 (s, 3H, CH_3_-thiazole).

### 2.4 Synthesis of AMP

The peptides temporin-L (FVRWFSRFLGRIL) and azide-temporin-L (K(N_3_)GGGFVRWFSRFLGRIL) were purchased from AAPPTec (Kentucky, USA), where K(N_3_) stands for a lysine residue modified with an azide group in its ε-amino group. The purified peptides were characterized by high-performance liquid chromatography and mass spectrometry.

### 2.5 Synthesis of Polyitaconate Derivatives Bearing Thiazolium Groups, PTTIQ

The incorporation of the thiazolium derivative synthesized in Section 2.3 into clickable biobased polymer P(PrI) was performed by CuAAC click chemistry. In a typical procedure, P(PrI) polymer bearing alkyne groups (0.150 g, 0.72 mmol), cationic thiazolium derivative functionalized with azide (M2Q) (0.496 g, 1.6 mmol), CuCl (7.2 mg, 0.073 mmol), and PMDETA (30 μL, 0.14 mmol) were dissolved in 5 mL of DMSO, and the mixture was stirred at room temperature for 24 h. Following that, the resulting cationic polymer (PTTIQ) was purified via dialysis and was subsequently recovered through lyophilization. (0.366 g, 61% yield). ^1^H NMR (400 MHz, DMSO-*d*_6_, δ, ppm): 10.02 (2H, *H*-thiazolium), 8.20 (2H, *H*-triazole), 5.05 (4H, O–CH_2_-triazole), 4.65 (4H, CH_2_–N), 4.08 (6H, N^+^CH_3_ thiazole), 3.55 (4H, C*H*_*2*_-thiazolium), 2.44 (6H, CH_3_-thiazolium), 2.00–1.00 (4H, CH_2_–CO and –CH_2_-chain).

### 2.6 Synthesis of Polyitaconate Derivatives Bearing Thiazolium Groups and Antimicrobial Peptide, Conjugate PTTIQ-AMP

The azide-temporin-L AMP was incorporated into the polymer chain by the CuAAC reaction via alkyne groups. P(PrI) polymer (0.010 g, 0.097 mequiv), peptide (0.020 g, 0.010 mmol), CuCl (1 mg, 0.01 mmol), and PMDETA (3.8 μL, 0.020 mmol) were dissolved in 5 mL of DMSO, and the mixture was stirred at room temperature for 24 h. Subsequently, M2Q (0.027 g, 0.087 mmol) was added to the reaction mixture, and the solution was stirred for another 24 h. After that, the conjugated peptide–polymer (PTTIQ-AMP) was purified by dialysis against deionized water and subsequently lyophilized (0.019 mg, 33% yield). ^1^H NMR (400 M Hz, DMSO-*d*_6_, δ, ppm): 10.70 (N*H*-Tryptophan, 10.02 (*H*-thiazolium), 8.4–7.7 (*H*-triazole and N*H* amide of peptide), 7.4 (aromatic protons of peptide) 5.05 (O–CH_2_-triazole), 4.65 (4H, CH_2_–N), 4.08 (6H, N^+^CH_3_ thiazole), 3.55 (4H, C*H*_2_-thiazolium), 2.44 (6H, C*H*_3_-thiazolium), 2.00–1.00 (4H, CH_2_–CO and -CH_2_-chain), 0.80 (*CH*_3_-valine, leucine, and isoleucine).

### 2.7 Preparation of Antimicrobial Functional Fibers via Electrospinning

Electrospinning solutions were prepared by dissolving 90 wt % of PLA and 10 wt % of polyitaconate derivative PTTIQ in a mixture of CHCl_3_/DMF (90/10 v/v) at a concentration of 20% w/w. We have selected the composition of the fiber mats based on previous works.^4,5^ PLA solutions were also prepared and used as a blank sample. These solutions were utilized to produce electrospun polymeric fibers by using a custom-made electrospinner with a horizontal configuration. The electrospinner featured a syringe needle connected to a high-voltage power source. Fibers mats were collected by utilizing a grounded aluminum foil collector positioned perpendicularly at a distance of 12 cm from the needle tip. The electrospinning process involved a flow rate of 1 mL h^–1^ and the application of a voltage of 16 kV for 30 min at room temperature. The resulting fibers were subsequently dried under vacuum at room temperature for 24 h and labeled as PLA/PTTIQ and PLA fiber mats.

### 2.8 Functionalization of PLA-Based Fibers by SSI

The PLA and PLA/PTTIQ fiber mats were impregnated with the AMP temporin-L using ScCO_2_ as a solvent in a commercially supplied device Speed SFE-4 (Applied Separations Inc., USA). Briefly, 6.6 mg of the corresponding fiber mat, 5 mg of AMP, and 0.2 mL of ethanol were placed in the high-pressure reactor. The chamber was closed and operated at 40 °C and 150 bar of pressurized CO_2_ for 3 h. Then, depressurization took place until atmospheric pressure. The mass of impregnated AMP was determined gravimetrically by measuring the weight of samples before and after impregnation. The impregnation yield (I) of PLA and PLA/PTTIQ fibers was calculated according to
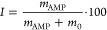
where *m*_0_ is the initial mass of fiber mats, and *m*_AMP_ is the mass of impregnated fibers with AMP.

### 2.9 Characterization

NMR spectra were acquired at room temperature using deuterated solvents on a Bruker AVANCE III HD-400AVIII spectrometer. Size exclusion chromatography (SEC) measurements were conducted using a Waters Division Millipore system, which was equipped with a Waters 2414 refractive index detector. The eluent used was DMF stabilized with 0.1 M LiBr (Sigma-Aldrich, >99.9%) at 50 °C and at a flow rate of 1 mL min^–1^. Poly(methyl methacrylate) standards (Polymer Laboratories Ltd.) were employed for calibration purposes. FTIR spectra were collected by utilizing a PerkinElmer Spectrum Two instrument that was equipped with an attenuated total reflection module. Dynamic light scattering and zeta potential measurements in distilled water at 25 °C were performed with a Zetasizer Nano series ZS instrument (Malvern Instruments Ltd.). To analyze the morphology of the electrospun fibers, scanning electron microscopy (SEM) was employed on previously gold-coated samples using a Philips XL30 microscope with an acceleration voltage of 25–10 kV.

### 2.10 Antimicrobial Assays

The antibacterial activities of the cationic polymer PTTIQ and AMP temporin L were evaluated by determining the MICs using the standard broth dilution method provided by the Clinical Laboratory Standards Institute (CLSI).^25^ Bacterial cells were grown on 5% sheep blood Columbia agar plates for 24 h at 37 °C. Then, fresh Mueller Hinton broth was used to adjust the bacterial concentration to 10^6^ cfu mL^–1^. Solutions of the polymer or AMP at a concentration of 20 mg mL^–1^ (1.9 and 11 mM, respectively) were also obtained in the Mueller–Hinton broth. In the subsequent step, 100 μL from each stock solution was dispensed into the first column of a 96-well round-bottom microplate, while the remaining wells were supplemented with 50 μL of broth. Starting with the first column, a polymer solution (50 μL) was diluted in a twofold serial manner across the remaining wells. Subsequently, 50 μL of the bacterial solution was added to achieve a total volume of 100 μL and a bacterial concentration of 5 × 10^5^ cfu mL^–1^. A positive control lacking the antimicrobial and a negative control lacking bacterial strains were also prepared. After incubation at 37 °C for 24 h, the MIC values against each strain were estimated by checking visually the absence of bacterial growth. All the tests were performed in three biological replicates.

To estimate the antimicrobial combinations, the fractional inhibitory concentration index (FICI) was calculated according to the checkerboard assay.^26,27^ In this assay, a broth dilution method was performed from the stock solutions of both antimicrobial compounds prepared as described below, starting from concentrations of 2 × MIC and using a bacterial suspension at a final concentration of 5 × 10^5^ cfu mL^–1^. In a 96-well round-bottom microplate, columns from 1 to 9 contain twofold serial dilutions of PTTIQ, while rows from A to G contain serial dilutions of AMP. Column 10 contains serial dilutions of PTTIQ alone, and row H contains serial dilutions of AMP alone, used to determine the MIC value of each compound. Column 11 was used as positive growth control without the antimicrobial compound, and column 12 was used as negative control, containing only the growth medium. The antimicrobial interactions were expressed as the FICI values, which are calculated as
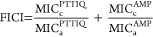
where MIC_c_ are the MICs of the combinations and MIC_a_ are the MICs of the antimicrobial alone.

The prepared fiber mats functionalized with the antimicrobial compounds (PTTIQ and/or AMP) were analyzed following the E2149-20 standard method^28^ from the American Society for Testing and Materials (ASTM) against *E. coli* and *E. faecalis* strains previously cultured for 24 h at 37 °C on 5% sheep blood Columbia agar plates. Next, the bacterial suspensions of 10^6^ cfu mL^–1^ were prepared in PBS. Next, fiber mats (1 mg) were introduced in sterile falcon tubes containing 0.1 mL of the tested inoculum and 0.9 mL of PBS (∼10^5^ cfu mL^–1^). Control experiments were conducted under two conditions: in the presence of PLA fiber without any antimicrobial compound and in the absence of mats entirely. The suspensions were subjected to shaking at 120 rpm for 24 h. Following that, the grown colonies were quantified using the plate counting method, and the reduction percentage was estimated in comparison to the control. The measurements were made, at least, in triplicate.

The release of the antimicrobial compounds from the PLA fibers to the medium was evaluated by the presence or absence of zones of inhibition around the sample (6 mm in diameter) when they are placed on agar plates inoculated with *E. coli* (∼10^5^ cfu mL^–1^) by the spread plate method and after incubation for 24 h at 37 °C.

## 3 Results and Discussion

### 3.1 Synthesis of Antimicrobial Biobased Polymers Derived from Itaconic Acid

In our previous study, we successfully developed biobased polymers derived from itaconic acid that incorporate triazolium and thiazolium groups, thus exhibiting antibacterial properties. The synthesis process involved multiple steps. Initially, a clickable polyitaconate was reacted with an azide-modified thiazole molecule, resulting in the formation of a polyitaconate polymer with side chains containing both thiazole and triazole groups via a cycloaddition reaction. Subsequently, an additional step involving N-alkylation was necessary to obtain the final cationic polymer, which contained thiazolium and triazolium groups (with a total of four cationic groups per monomeric unit).

**Figure 1:**
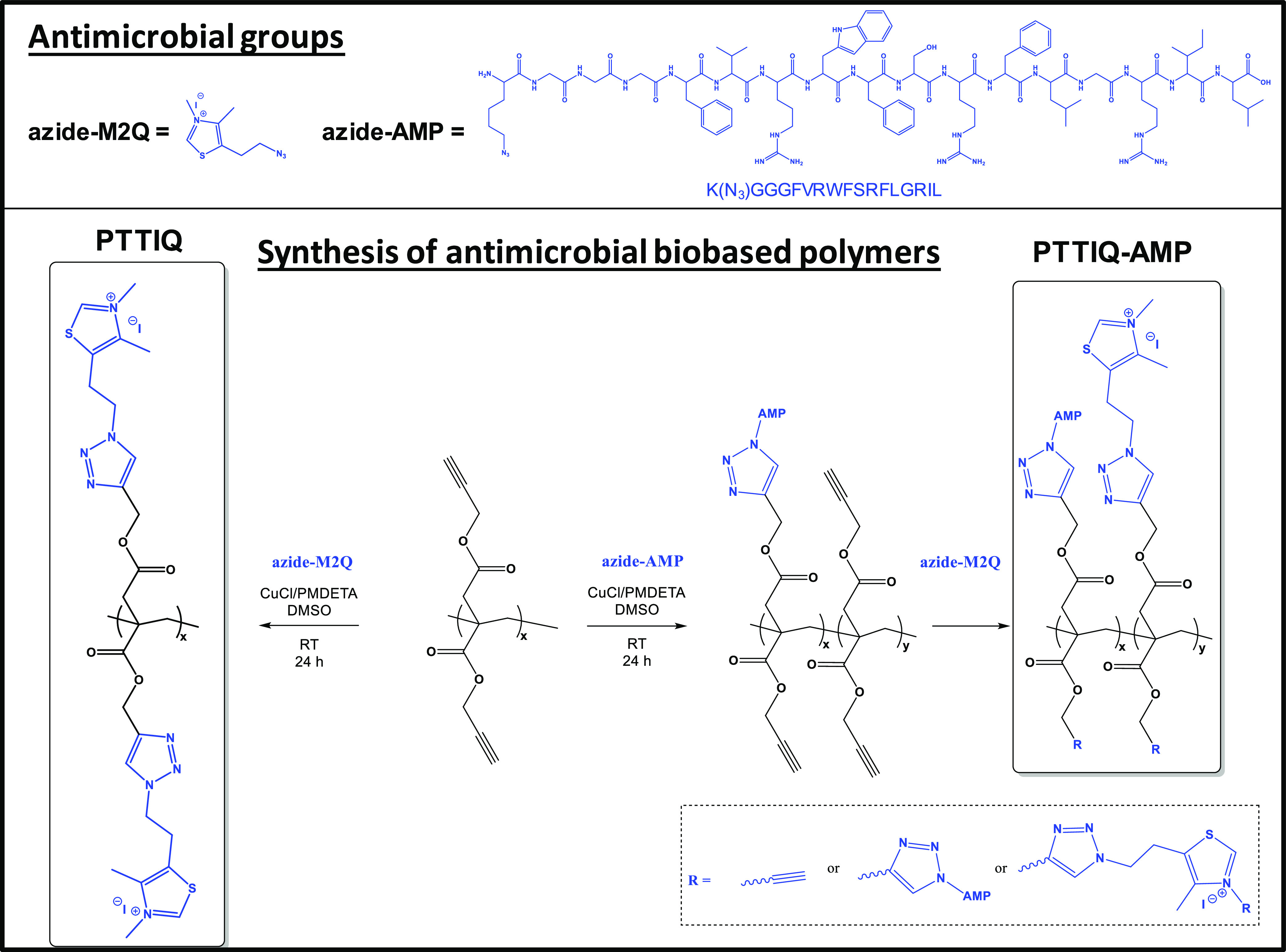
Synthesis of cationic biobased polyitaconate bearing thiazolium groups (PTTIQ) and of the conjugated structure, biobased polyitaconate bearing thiazolium and AMP (PTTIQ-AMP).

In this current work, we improved the synthetic approach by quaternizing the azide-modified thiazole molecule prior to the click chemistry reaction. This modification enabled the direct formation of the cationic polyitaconate with the cationic charge selectively located on the thiazolium moieties. Simultaneously, we explored the incorporation of other functionalities into the clickable polymer to synthesize antimicrobial materials with a combined action. Specifically, we successfully attached the AMP temporin L to the polyitaconate, utilizing the thiazolium group for conjugation (Figure 1).

**Figure 2:**
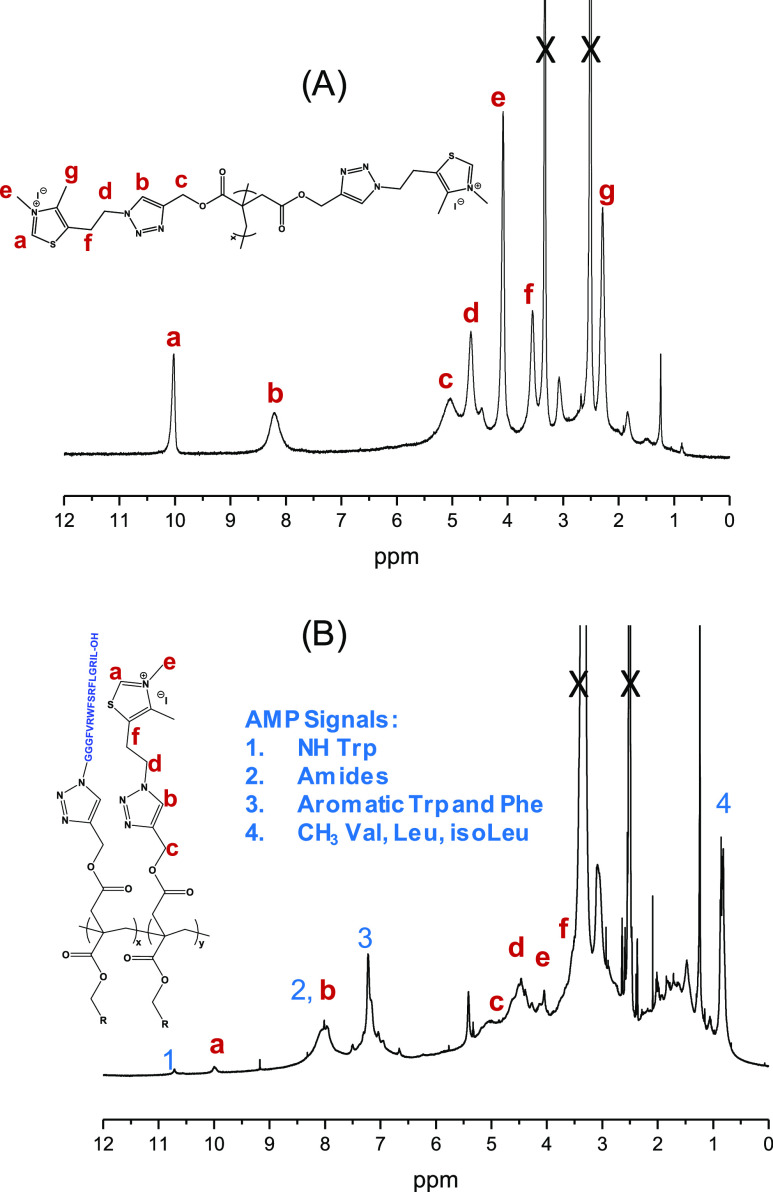
^1^H NMR spectra of (A) PTTIQ and (B) PTTIQ-AMP biobased polymer.

**Figure 3:**
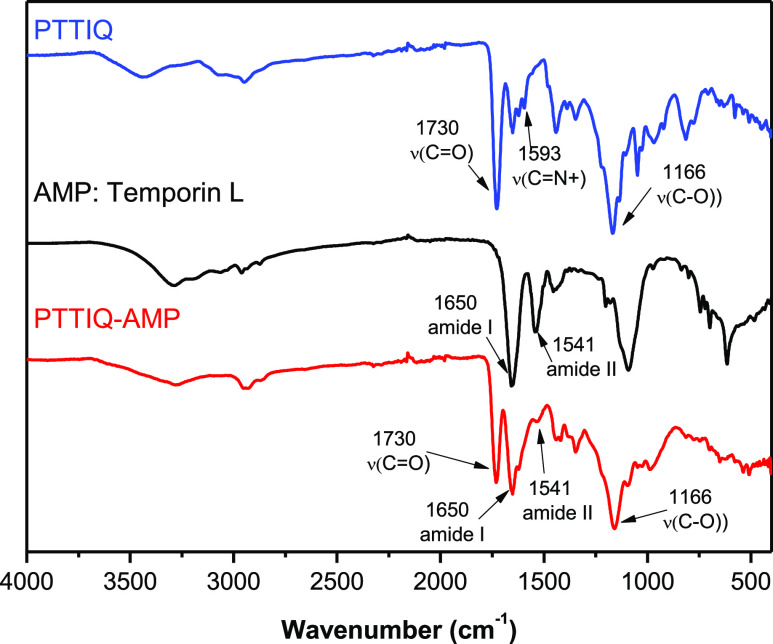
FTIR spectra of PTTIQ, temporin L peptide, and the PTTIQ-AMP conjugated polymer.

Next, we characterized the synthesized polymers by nuclear magnetic resonance (Figure 2). The ^1^H NMR spectra of the synthesized polymers, in which all signals can be assigned to the targeted polymer structures, were successfully obtained. Based on the intensity of the NMR signals, the composition of the obtained conjugated polymer (PTTIQ-AMP) was estimated to be 70 mol % of M2Q thiazolium and 30 mol % of AMP, thus yielding a higher content of peptide than the content used initially for the synthesis (N_3_-M2Q/N_3_-AMP: 90/10). This can be attributed to the steric hindrance of AMP, which may have hindered the subsequent reaction of the unreacted alkyne groups from the polyitaconate chain, consequently affecting the attachment of all thiazolium groups. FTIR spectra also corroborate the incorporation of both antimicrobial components to the biobased polymer (Figure 3). In the FTIR spectrum of PTTIQ, the bands at 1730 cm^–1^ assigned to υ(C=O), 1593 cm^–1^ corresponding to the υ(C=N+), and 1166 cm^–1^ due to C–O stretching vibration were observed, whereas in the PTTIQ-AMP FTIR spectrum, new bands appear associated with the AMP, temporin L, in addition to the bands of the thiazolium groups. The main bands associated with the peptide appear at 1650 cm^–1^ (amide I, mainly related with the C=O stretching vibration) and at 1541 cm^–1^ (amide II, N–H bending), indicating the presence of amide bonds in the peptide’s backbone.

### 3.2 Antimicrobial Properties of Biobased Polymers

Subsequently, the antimicrobial activity of the prepared polymers with thiazolium groups, PTTIQ, and with thiazolium and AMP conjugate, PTTIQ-AMP, was assessed by determining the MIC values against *P. aeruginosa*, *E. coli*, *S. aureus*, *E. faecalis*, and *C. albicans* strains. We synthesized the polymer–peptide conjugate (PTTIQ-AMP) with the goal of achieving superior antimicrobial activity, stability, and solubility. Conjugating AMP to polymers often provides protection against protease degradation and, in some cases, can reduce the cytotoxic effects and increase the antibacterial activity.^15^ For instance, it has been reported that polylysine AMPs attached to chitosan generated significant damage to bacterial membranes.^29,30^ As shown in Table 1, the cationic polyitaconate bearing thiazolium groups (PTTIQ) is effective against Gram-positive bacteria with MIC values of 64 μg mL^–1^ and has moderate activity against the fungus *C. albicans*, whereas it is not active against Gram-negative bacteria. In contrast, the AMP temporin L has a broad antimicrobial spectrum, effectively targeting both Gram-positive and Gram-negative bacteria and *C. albicans*. However, upon conjugation of the thiazolium group with the AMP within the polyitaconate chains (PTTIQ-AMP), significantly higher MIC values were observed compared to both the corresponding polymer with only thiazolium groups and the standalone temporin L peptide. This observation can be attributed to the aggregation of the polymeric chain, leading to reduced solubility in this case, and consequently limiting the availability of functional antimicrobial groups for interaction with microorganisms. In effect, zeta potential measurements indicated a significant decrease in the value of the PTTIQ-AMP conjugate (31 ± 5 mV) compared to the values obtained for the cationic polymer (52 ± 5 mV) and the peptide (41 ± 5 mV). These findings could be indicative of an aggregation process occurring within the PTTIQ-AMP conjugate, which could hinder the positive charges and then reduce the antimicrobial efficacy.

**Table 1:** MIC Values of PTTIQ, Temporin L AMP, and PTTIQ-AMP

	MICs (μg mL^–1^)					MICs in combination (μg mL^–1^)	
Sample	*C. albicans*	*P. aeruginosa*	*S. aureus*	*E. coli*	*E. faecalis*	*E. coli*	*E. faecalis*
PTTIQ	125	>500	64	500	64	250	64
AMP	32	125	4	16	8	8	8
PTTIQ-AMP	500	>500	125	>500	125		

For this reason, we decided to assess the impact on the antimicrobial activity of the combination of the cationic polymer PTTIQ and the AMP temporin L without conjugation in the same polymeric structure. The FICI values of the combination (PTTIQ-AMP) against the Gram-positive *E. faecalis* and Gram-negative *E. coli* bacterial strains were determined using the checkerboard method.

In this assay, the combination is considered synergistic when FICI ≤0.5, additive if 0.5 < FICI ≤ 1, indifferent when 1 < FICI ≤ 2, and antagonistic when FICI > 2.^31^ The FICI against *E. faecalis* and *E. coli* was found to be 2 and 1.5, respectively; thus, an indifferent effect is observed, meaning that a combination of cationic polymer and AMP has an effect identical to that of the most active constituent. In principle, the mechanism of action of the cationic polymer and temporin L is mainly through interactions with the negatively charged bacterial membranes leading to insertion, destabilization, and consequent disruption of the membrane.^32^ Temporin L’s ability to damage and penetrate the bacterial membrane is associated with its highly stable secondary amphipathic α-helical conformation. Although it has been studied that synergistic interactions could be probable with antimicrobial agent combinations that act on a similar manner,^33,34^ in this case, the combination had no synergistic effect.

### 3.3 Preparation of Antimicrobial Fiber Mats via Electrospinning

**Figure 4:**
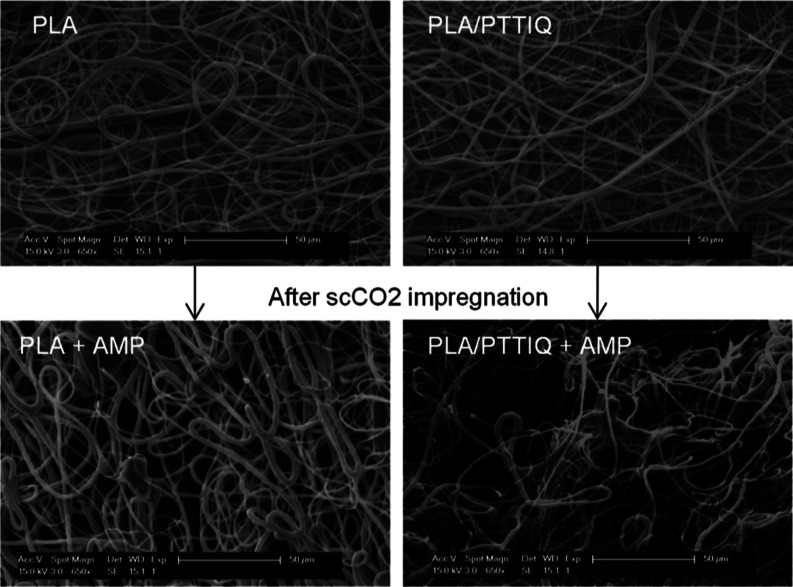
SEM images of prepared fiber mats before (PLA and PLA/PTTIQ) and after (PLA + AMP and PLA/PTTIQ + AMP) scCO_2_ impregnation with AMP.

Subsequently, the antimicrobial biobased polymer and the AMP temporin L were employed to confer activity to fiber mats based on PLA. Due to the significantly low antimicrobial activity exhibited by the PTTIQ-AMP conjugated structure, this polymer was excluded from the experimental investigations. Despite the combination of PTTIQ and temporin showing indifferent effects when exposed to the bacterial suspension medium, we proceeded to test the combination of PTTIQ and AMP temporin L, which was immobilized in PLA fibers. This decision was based on the understanding that the availability of both antimicrobial compounds to interact with the bacteria might vary due to differences in diffusion, migration, and release processes. Initially, PLA fibers and PLA/PTTIQ (90/10 wt %) fibers were directly electrospun from solutions containing CHCl_3_ and DMF. Next, we analyzed the morphology of the obtained fibers in both cases (Figure 4), in which it is evident that the incorporation of 10 wt % of PTTIQ did not induce changes in the resulting fibers, albeit a slight reduction in diameter is observed, from 1.6 ± 0.6 μm for PLA to 1.2 ± 0.5 μm for PLA/PTTIQ fibers.

In a second step, AMP temporin L was incorporated into both fiber mats (PLA and PLA/PTTIQ) by SSI. This technique allows the use of a low amount of peptide to impregnate a relatively high volume of solid sample without the use of any toxic organic solvent under moderate experimental conditions. It is well-known that both the operating conditions and the structure of the polymer strongly affect the impregnation yield. In this study, we compared the SSI efficiency of PLA and PLA/PTTIQ fibers by applying fixed conditions of 40 °C and 150 bar for 3 h. The AMP impregnation yields were determined gravimetrically, 27 and 6%, for PLA and PLA/PTTIQ, respectively. We hypothesize that the lower impregnation yield obtained with the PLA/PTTIQ fibers in comparison to that obtained with the PLA fibers is associated with the presence of the cationic polymer that might hinder the incorporation of the peptide by electrostatic repulsion or because the cationic polymer impedes the swelling of the PLA fibers. SEM images of the impregnated fibers (Figure 4) confirm that, under the chosen temperature and pressure conditions, the scCO_2_ impregnation process does not significantly alter the morphology of the fibers, although some fiber breakages are observed. However, both fiber mats exhibit an increase in diameter, indicating the swelling effect induced by carbon dioxide on the polymeric fibers. The diameter of the PLA + AMP fibers, 2.5 ± 0.9 μm, is larger than that of PLA/PTTIQ + AMP impregnated fibers, 2 ± 1 μm. Nonetheless, the swelling percentage relative to the nonimpregnated fibers is slightly lower for the latter case. Therefore, the low loading yield observed in PLA/PTTIQ may be attributed to repulsive interactions with the cationic polymer.

**Figure 5:**
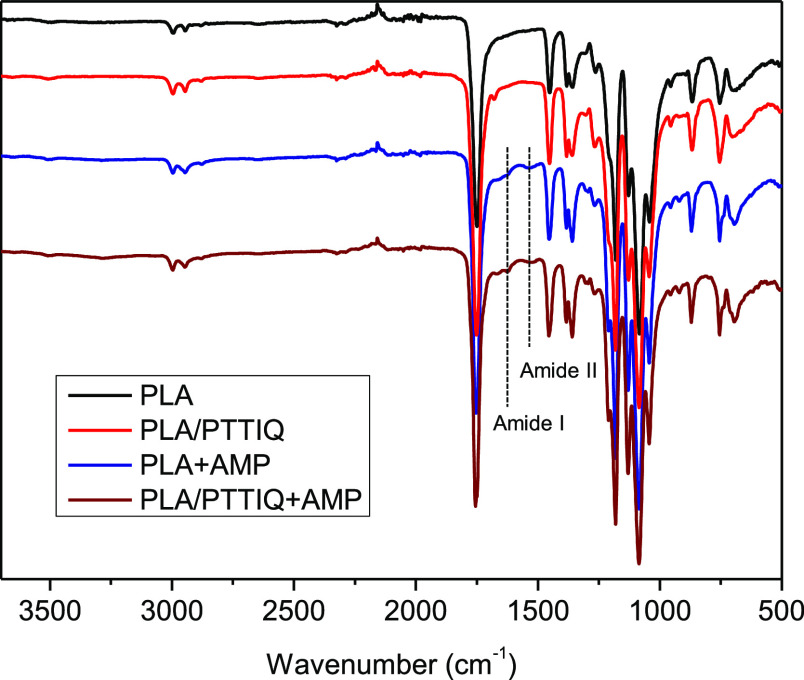
FTIR spectra of nonimpregnated fibers (PLA and PLA/PTTIQ) and impregnated fibers with AMP (PLA + AMP and PLA/PTTIQ + AMP).

FTIR spectra were recorded to evaluate the chemical structures of PLA and PLA/PTTIQ fibers before and after peptide impregnation using scCO_2_ (Figure 5). The spectra of PLA and PLA/PTTIQ reveal the characteristic absorption bands for PLA, at 1756 cm^–1^, due to the stretching of carbonyl group—C=O, and bands at 1450 cm^–1^ assigned to the CH_3_ asymmetric bending vibration, at 1183 cm^–1^ corresponding to the C–O–C stretching vibration, and at 1083 cm^–1^ associated with C–O stretching. In the FTIR spectrum of the fibers, several new bands appeared after impregnation. The presence of the AMP is confirmed by the bands associated with amide I and amide II, at 1650 and 1541 cm^–1^, respectively.

After confirming the success of the AMP impregnation, we investigated the antimicrobial activity of PLA and PLA/PTTIQ fibers impregnated with the AMP (PLA + AMP and PLA/PTTIQ + AMP) in comparison with the corresponding nonimpregnated samples against *E. coli* and *E. faecalis*. The results obtained from the tested fibers, using the ASTM E2149 method, which determines the percentage of bacterial reduction after incubation under dynamic conditions related to an initial inoculum, are summarized in Table 2.

**Table 2:** Antimicrobial Activity of Nonimpregnated Fibers (PLA and PLA/PTTIQ) and of Impregnated Fibers with AMP (PLA + AMP and PLA/PTTIQ + AMP) against *E. coli* and *E. faecalis*

	*E. coli*		*E. faecalis*	
Sample	Bacterial reduction (%)	log reduction	Bacterial reduction (%)	log reduction
PLA/PTTIQ	97	1.58	96	1.39
PLA/PTTIQ + AMP	99.999	5	99.999	5
PLA + AMP	99.999	5	99.999	5
PLA/PTTIQ^a^	83	0.77	77	0.63
PLA/PTTIQ + AMP^a^	84	0.78	92	1.10
PLA + AMP^a^	0	0	45	0.26

a Results obtained in a second cycle of the antibacterial test.

It is evident that the fibers impregnated with the AMP temporin L exhibit high antimicrobial activity, achieving a log reduction up to 5 against both bacterial strains. In contrast, the fibers containing only the cationic polyitaconate yield log reductions of 1.58 and 1.39 against *E. coli* and *E. faecalis*, respectively. These findings confirm the potential of temporin L as an antimicrobial agent in PLA-based fibers.

Subsequently, the antibacterial efficacy of the fibers was retested in a second cycle. The fiber mats were subjected to sequential washing with ethanol, PBS, and water and subsequently dried overnight in a vacuum oven. The antibacterial test against both bacterial strains was then repeated as described above. The fibers experienced a significant decrease in activity, which can be attributed to the leaching of the antimicrobial component during the first antimicrobial test, resulting in a reduction of the available antimicrobial agent for the second cycle (Table 2). This effect is more pronounced in fiber mats impregnated with AMP (PLA + AMP and PLA/PTTIQ + AMP), suggesting that the loss/leaching of the AMP during incubation is greater than that of the cationic polyitaconate, probably because the scCO_2_ impregnation of AMP occurs mostly at the surface of the fiber mats. In contrast, the cationic polyitaconate mostly remains embedded in the fibers, maintaining its antimicrobial performance likely due to its higher molecular weight and lower diffusion capacity.

**Figure 6:**
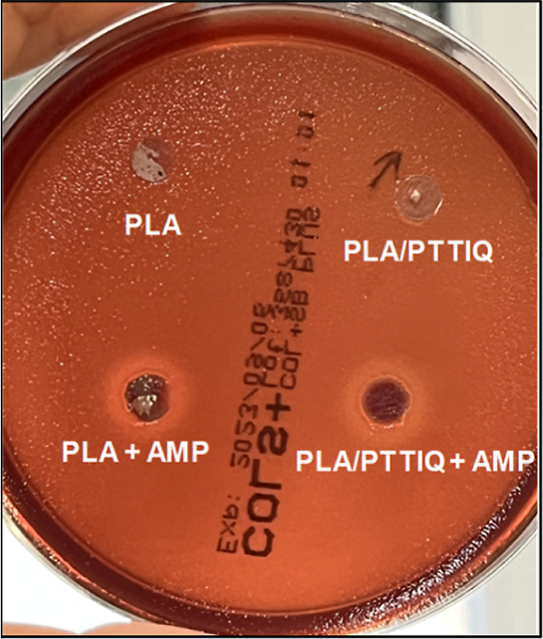
Photographs of disk diffusion assays on agar plates cultivated with Gram-negative *E. coli* of nonimpregnated fibers (PLA and PLA/PTTIQ) and impregnated fibers with AMP (PLA + AMP and PLA/PTTIQ + AMP).

Disk diffusion testing was performed to confirm the release of the AMP from the fiber mats. As can be seen in Figure 6 for the test with Gram-negative *E. coli*, only the fibers mats impregnated with AMP (PLA + AMP and PLA/PTTIQ + AMP) form an inhibition zone, revealing the diffusion of the peptide out of the fibers.

Nevertheless, as PLA is a biodegradable material, the cationic polymers and the AMP could be released from the fibers during its degradation process.^35,36^

## 4 Conclusions

In conclusion, we have shown that the combination of a cationic biobased polyitaconate incorporating thiazolium moieties with AMP temporin L did not improve the antibacterial activity against various microorganisms. In fact, an indifferent effect was observed, and even a negative effect was noticed when temporin L was covalently conjugated as a side chain of the cationic polyitaconate. However, the clickable biobased polymer has demonstrated its suitability and versatility as a platform for specific tethering of bioactive groups, enabling the development of multifunctional systems with the aim of identifying synergistic combinations.

On the other hand, the incorporation of both the cationic polymer and temporin L into PLA fibers has shown improved antibacterial performance. This enhancement can be attributed to their different diffusion capacities and leaching behaviors, which contribute to the more effective and sustained release of antimicrobial agents.
